# Long-Term Performance of Passive Volatile Organic Compounds (VOCs) Samplers for Indoor Air

**DOI:** 10.3390/environments12080267

**Published:** 2025-07-31

**Authors:** John H. Zimmerman, Brian Schumacher, Christopher C. Lutes, Brian Cosky, Heidi Hayes

**Affiliations:** 1 Watershed and Ecosystem Characterization Division, Center for Environmental Measurement & Modeling (CEMM), Office of Research and Development (ORD), U.S. Environmental Protection Agency (EPA), 109 TW Alexander Dr., Research Triangle Park, NC 27711, USA; 2 Ecosystem Processes Division, Center for Environmental Measurement & Modeling (CEMM), Office of Research and Development (ORD), U.S. Environmental Protection Agency (EPA), 960 College Station Road, Athens, GA 30605, USA; 3 Jacobs, 1999 Bryan St., Ste. 1200, Dallas, TX 75201, USA; 4 Eurofins Environment Testing Northern California, LLC, Folsom, CA 95630, USA

**Keywords:** passive sampling, vapor intrusion, PCE, chloroform, TCE, indoor air

## Abstract

The reliability of passive samplers in measuring volatile organic compounds (VOCs) in indoor air depends on whether the uptake rate is constant given the environmental conditions and sampler exposure duration. The first phase of this study evaluated the performance of charcoal-based, solvent-extracted passive samplers (e.g., Radiello^®^ 130 passive samplers with white diffusive bodies) over exposure periods ranging from 1 week to 1 year in a test house with known vapor intrusion (VI). Chloroform %Bias values exceeded the ±30% acceptance criterion after 4 weeks exposure. Benzene, hexane, and trichloroethylene (TCE) concentrations were within the acceptance criterion for up to three months. Toluene and tetrachloroethylene (PCE), the two least volatile compounds, demonstrated uniform uptake rates over one year. In the second phase of this study, testing of the longer exposure times of 6 months and 1 year were evaluated with three additional passive samplers: Waterloo Membrane Sampler^™^ (WMS^™^), SKC 575 with secondary diffusive cover, and Radiello^®^ 130 passive samplers with yellow diffusive bodies. The SKC 575 and Radiello^®^ 130 passive samplers produced acceptable results (%Bias ≤ 30%) over the 6-month exposure period, while the WMS^™^ sampler results favored petroleum hydrocarbon more than chlorinated solvent uptake. After the 1-year exposure period, the passive sampler performances were acceptable under specific conditions of this study. The results suggest that all three samplers can produce acceptable results over exposure time periods beyond 30 days and up to a year for some compounds.

## 1. Introduction

The objective of this study was to evaluate volatile organic compound (VOC) concentrations using passive samplers beyond 30 days and potentially extending the exposure period to a full year. The few published studies evaluating sampling intervals greater than 30 days are largely focused on measuring benzene, toluene, ethylbenzene, and xylenes (BTEX) [1,2]. The stability of chlorinated compounds on sorbents in the presence of humidity and the variability of the sampler uptake rate past 30 days are not well understood for any of the passive samplers under consideration for this study. The sorbent selection, sampler geometry, and target chemical’s volatility may impact the application of passive samplers during extended deployment/exposure periods. Vapor intrusion (VI) is the migration of subsurface vapors, including radon and VOCs, from the subsurface of a building to indoor air. Current standard practices for indoor air VOC monitoring in the US include the use of negatively pressurized, ultra-clean, passivated, stainless steel canisters for collecting samples to be analyzed by methods TO-14 and TO-15 [3,4]. Method TO-15 specifies an audit accuracy of ±30% and a replicate precision of 25% as performance criteria.

Active (or pumped) sorbent methods (e.g., TO-17 [5]) have also been published by the US Environmental Protection Agency (US EPA) for VOC measurements in ambient air. However, in these methods, air samples are actively collected over 1 h using a sample pump with sampling rates from 17 mL/min to 67 mL/min, yielding total sample volumes between 1 and 4 liters (L). Sampling intervals can be extended beyond 1 h; however, care must be taken to ensure breakthrough volumes are not exceeded. Given the minimum pump flow rate cited in TO-17 of 17 mL/min, the practical upper limit for chlorinated VOCs using a multibed thermal desorption sorbent tube is on the order of 10 L to 20 L for select VOCs yielding a corresponding maximum collection period of 8 to 24 h [6].

European agencies have developed standard methods for passive sampling for VOCs applicable to a range of concentrations and durations that are similar to those discussed by the study in this paper for indoor and ambient air quality, as well as vapor intrusion investigations. This material and other basic information on passive samplers have been summarized in an Engineering Issue Paper prepared by US EPA’s Office of Research and Development [7].

One way to lower the detection limits and control day-to-day variability when evaluating chronic or sub-chronic exposures is to sample over a longer period. This approach would provide a lower detection limit, be cost-effective, and result in a time-integrated composite sample. Laboratory and field evaluations with such an approach for ambient and indoor air applications have been published and show promising results for sampling durations of up to 14 days. Exposure of badge-type charcoal-based passive samplers to controlled atmospheres of 10 to 200 ppb benzene, toluene, and m-xylene showed a more consistent uptake rate when deployed for 14 days [8]. A field study published by Begerow et al. showed comparability between two charcoal-based passive sampler geometries, badge and tube-style, for 4-week indoor and outdoor air samples [9]. Field evaluations were also conducted using radial charcoal-based and thermal desorption Radiello^®^ samplers to determine performance over a 14-day period. Ambient BTEX measurements using Radiello^®^ samplers compared well to active sorbent sampling results [10].

A field study at Orion Park, Moffett Field, in California compared measurements of VOCs by Method TO-15 [4] to three different radial and axial tube-type sorbent systems:

1. Radial: activated charcoal (with carbon disulfide (CS_2_) extraction: gas chromatography–mass spectrometry [GC/MS]);
2. Radial: carbograph 4 (TO-17 [5]: thermal desorption [TD] GC/MS);
3. Axial: chromosorb 106 thermal desorption tube (TO-17 [5]: TD GC/MS).

Performance for the two radial samplers was superior to the axial method [11,12]. Testing was also performed at the Wheeler building in Indianapolis comparing Summa canisters to Radiello^®^ solvent-extracted samplers. The Wheeler building is a complex, multiuse, multiunit 100,000 square foot building that has been previously tested by US EPA [11–13]. Formerly a factory from 1911 to 1995, the industrial slab-on-grade and basement building was renovated with a theater, office space, and artist galleries, including live/work lofts with 35 separate heating, ventilation, and air conditioning (HVAC) zones.

Across the two sites, the Radiello^®^ solvent-extracted sampler concentrations of chlorinated compounds, such as PCE and TCE, showed good agreement with TO-15 Summa canister results and had recoveries generally ranging from 70 to 100% and precision % differences less than 5%. Agreement was poor for polar compounds, including ethanol, methyl ethyl ketone, and acetone, whereas the Radiello^®^ solvent-extracted samplers recovered between 5 and 30% when compared to the TO-15 Summa canister results. Radiello^®^ thermal desorption sampler results correlated well (*r*^2^ = 0.85) with the TO-15 Summa canister results but had TCE concentrations about half of those found in the Summa canisters. The lower TCE concentrations suggest that a 2-week exposure time was too long for the Radiello^®^ thermally desorbed samplers [11,12].

McAlary et al. performed an extensive comparison of the performance among five different passive samplers in the laboratory and field sites [14,15]. In the laboratory, they found that passive samplers can provide concentration measurements with accuracy (mostly within a factor of 2) and precision (RSD < 15%) comparable to conventional Summa canister samples and US EPA Method TO-15 analysis when the passive samplers were exposed for 30 min [14]. Similar results were found when the same five samplers were exposed for up to 11.7 days in the field, where soil vapor concentrations were within a factor of 2 and precision was comparable to active sampling using Summa canisters [15].

The objective of this study was to evaluate VOC concentrations using passive samplers beyond 30 days, potentially extending the exposure period to a full year. The reliability of passive samplers in measuring VOC concentrations largely depends on whether the uptake rate is constant through time given the environmental conditions and the exposure period. Prolonged exposure of passive samplers can result in reduced net uptake rates due to back diffusion or loss of sorptive capacity. To evaluate the performance of charcoal-based, solvent-extracted, passive samplers over periods ranging from 1 week to 1 year, the VOC concentrations measured for extended time intervals were compared with the average concentrations measured concurrently over shorter time segments. The shortest interval used in this comparative study was 1 week.

## 2. Materials and Methods

The study was conducted in two phases. The first phase was an intensive year-long study looking at the Radiello^®^ 130 passive sampler performance on a weekly, bi-weekly, monthly, quarterly, and annual frequency. As a result of the findings from the first phase, a second-phase long-term study was conducted to examine the effectiveness of the three different passive samplers for exposure periods of 6 months and 1 year.

### 2.1. Study Site

**Figure 1.**
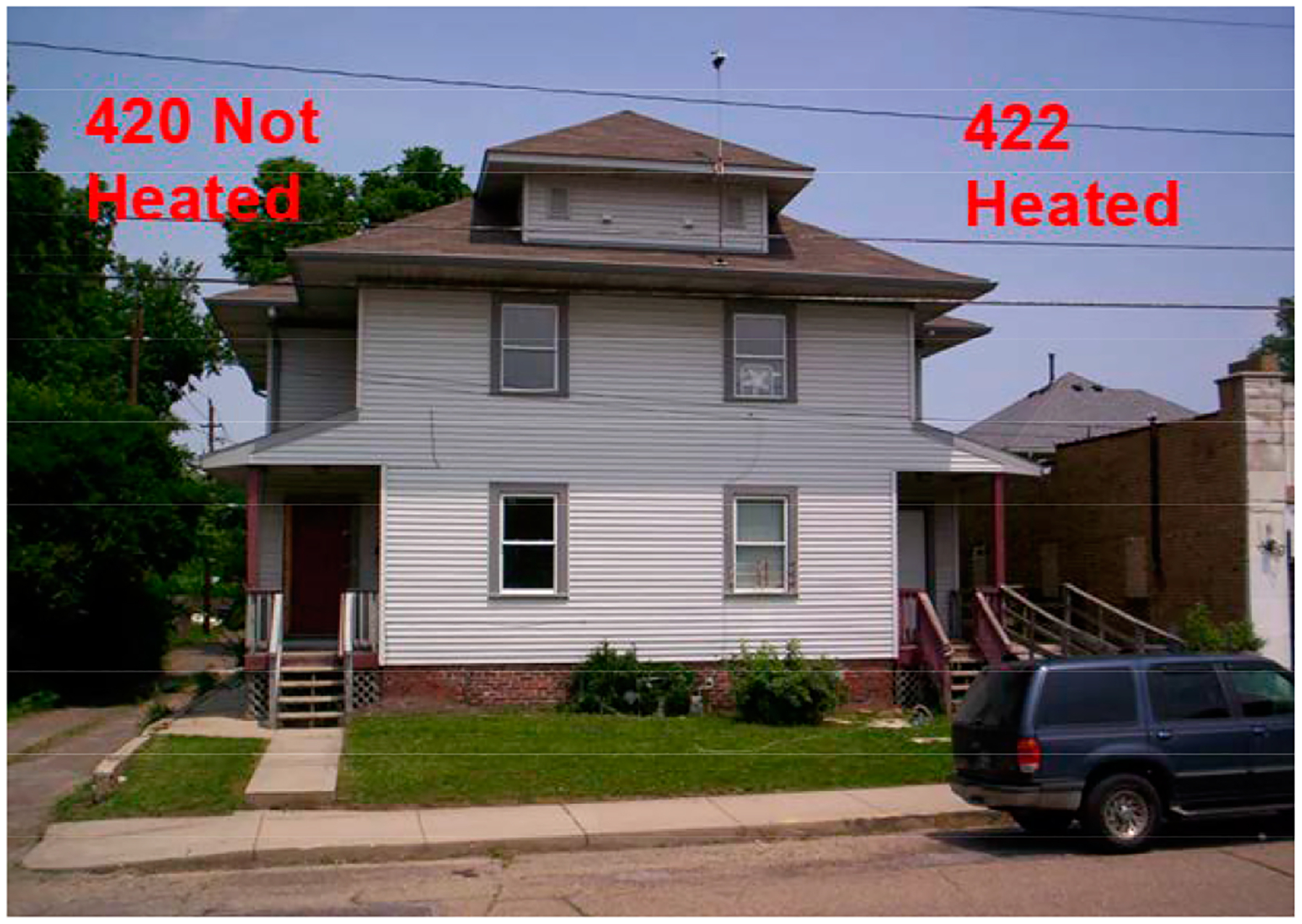
Street view of Indianapolis test house (Photo courtesy of US EPA).

The test house is a vacant residential duplex in the Mapleton Fall Creek neighborhood of Indianapolis, IN [16]. The test house (Figure 1) is an early twentieth century duplex built before 1915. Based on the mirrored floor plans of the two sides, it is likely that the house was always a duplex. The construction is a two-story wood frame (with attic) on a brick foundation with a poured concrete basement floor. Due to extensive vandalism prior to the start of the study commencing, a gas-fired forced air furnace unit was installed only on one side of the duplex. The test house has no central air conditioning system to mimic typical tenant usage; therefore, window-mounted air conditioning units were installed. The test house was unfurnished and operated as if occupied, with seasonal adjustments to temperatures being made by a field scientist, although heat was available only on one side of the duplex. As the test house was predominantly vacant, anthropogenic inputs of VOCs were not a concern.

### 2.2. Mitigation History

The Indianapolis test house was not mitigated during the phase 1 study [16]. Between the phase 1 and phase 2 studies, a subslab depressurization system was installed and periodically operated [17]. During phase 2, the mitigation system was off for the first 6 months and on for the second 6 months of the study.

### 2.3. Sample Collection

For phase 1, indoor air passive sampling was done with Radiello^®^ 130 samplers with white diffusive bodies. The Radiello^®^ 130 passive samplers were placed on wooden clothes drying racks for durations of 7, 14, 28 (monthly), 91 (quarterly), 182 (semiannually), and 364 (annual) days. On both sides of the Indianapolis test house, racks were placed in the first-floor center room and in the northern and southern sections of each basement. In addition, a protected outdoor location, on a telephone pole near the house, was installed to accommodate the passive samplers and to act as an ambient air location.

For phase 2, three different passive samplers were deployed to compare their efficiencies among sampler types over periods of 6 months and 1 year. The sampler types compared at the Indianapolis test house were these: (a) Radiello^®^ 130 radial sampler with a yellow diffusive body, which has a longer diffusive path length due to its smaller average pore size and a thicker diffusive membrane than the white diffusive body, which results in a lower sampling rate; (b) SKC 575 badge sampler with charcoal-based sorbent; and (c) WMS^™^, which is a permeation passive sampler in which the uptake of VOCs is controlled by a polydimethylsiloxane membrane and the VOCs are collected onto a charcoal-based sorbent bed. The WMS^™^ has the lowest sampling rates of the three samplers tested, with rates on the order of 2 to 5 times less than the SKC 575 badge and 5 to 20 times less than the Radiello^®^ 130 passive sampler with the yellow diffusive body. Specifications of the three types of passive samplers used in this study are provided in Table 1. Manufacturer’s guidelines and standard operating procedures for each of these passive samplers can be found at Radiello [18], SKC 575 [19], and WMS^™^ [20].

The selected samplers and the control Radiello^®^ 130 passive samplers with the white diffusive bodies were each hung on the wooden racks in the 422 first floor center room, 422 basement south section, 420 basement south section, and at the ambient location. The control Radiello^®^ 130 passive samplers with the white diffusive bodies were collected after 7-day exposure periods. Individual weekly results were used to identify peak VOC concentrations that should be reflected in the long-term sample results.

### 2.4. Sample Analysis and Quality Assurance

All passive samplers were analyzed by spiking internal standard and surrogate solution onto the sorbent and adding 2.0 mL of CS_2_ directly to each storage vial or the sampler housing. The vial or sampler was then placed on a shaker for approximately 30 min, and the extract was transferred to an autosampler vial for analysis by GC/MS by US EPA Method 8260 [22], in synchronous SIM/Scan mode with a 6% cyanopropyl phenyl, 94% polydimethylsiloxane, 30 m × 0.25 mm × 1.4 um column. Field blanks, trip blanks, and laboratory blanks were used to evaluate false positives and/or high bias due to transport, storage, sample handling, and sorbent contamination.

#### 2.4.1. Radiello^®^ 130

The uptake rates used to generate sample concentrations were published by the Radiello^®^ manufacturer, Fondazione Salvatore Maugeri (Padova, Italy), based on measurements made in a standard atmosphere chamber [18]. The rates were corrected for the average temperature recorded over the sampling duration using the equation:

(1) $${\text{Q}}_{\text{K}}={\text{Q}}_{298}{\left(\text{K}/298\right)}^{1.5}$$

where K is the measured temperature in degrees Kelvin, Q_K_ is the uptake rate at temperature K, and Q_298_ is the published reference rate at 298 K.

Evaluation of the passive sampler performance over the exposure period was determined by comparing the numerical average of the shorter time segments (e.g., 1 week and 2 weeks) to the concurrent integrated measurement (e.g., biweekly and monthly). For each interval evaluated, the relative percent difference (%Bias) was calculated using the following equation:

(2) $$\%\text{Bias}=({\text{C}}_{\text{A}}-{\text{C}}_{\text{I}})/(({\text{C}}_{\text{A}}+{\text{C}}_{\text{I}})/2)\times 100\%$$

where C_A_ = average concentration of the shorter exposure period sample and C_I_ = measured concentration of the integrated sample over the longer exposure period.

A positive %Bias indicated the average concentration of the shorter duration measurements was higher than the longer integrated sample concentration. A negative %Bias indicated that the shorter duration measurement underestimated the actual vapor concentration. The acceptance criterion to demonstrate equivalency between concentrations and durations was ±30%. If the reported concentration was a nondetect, the %Bias calculation was performed using half of the reporting limit for the corresponding concentration.

Field blanks were collected using a blank Radiello^®^ 130 cartridge from the media sample batch sent to the field from the laboratory. The cartridge was removed from the sealed storage vial and transferred to the diffusive housing in a similar manner to sample deployment. The cartridge was then immediately removed from the housing, returned to the storage vial, and sealed for shipment back to the laboratory with the field samples. In general, a field blank was collected with each shipment to the laboratory. A total of 47 field blanks were submitted over the duration of the project.

Blank Radiello^®^ 130 cartridges from the media batches were also assigned as trip blanks, measures of exposure during transportation to the laboratory. The cartridge was not opened or removed from the storage vial but was sent back to the laboratory along with the field samples. There were 22 trip blanks submitted for analysis.

In the case of the laboratory blank, a Radiello^®^ 130 cartridge was extracted with each analytical batch to measure background from the sorbent and the extraction process. A total of 73 unique laboratory blanks were analyzed and reported over the duration of the project. To assist in data interpretation, all blank and field sample results were evaluated down to the method detection limit (MDL). The results of the field, trip, and laboratory blanks for the Radiello^®^ 130 sampler are summarized in Tables A1–A3. The number of blanks with detections above the reporting limit (RL) and MDL are tabulated. Summary statistics were then calculated on this subset of positive detections.

Hexane and toluene were commonly detected in the field, trip, and laboratory blanks above the MDL. In the case of the field blanks, several had concentrations above the RL for hexane and toluene. All detections in the trip and laboratory blanks were below the RL but above the MDL. Positive biases for benzene, hexane, and toluene were anticipated for the daily Radiello^®^ 130 samples due to the blank levels. Sample concentrations were not adjusted for concentrations detected in any blank. For the daily passive samples, the average mass collected on the blank sorbent was 0.11, 0.10, and 0.04 μg for benzene, hexane, and toluene, respectively. A positive bias was expected for hexane for the weekly samples as well because the mass collected in the samples was generally less than 10 times the associated blank levels. Blank levels of toluene were not significant when evaluating the weekly samples because the mass collected in the samples was generally greater than 10 times the associated blank levels. Longer duration samples would normally collect more mass and thus would not be significantly affected.

No detections of chloroform or cis-1,2-dichloroethene (cis-1,2-DCE) were measured in any of the blanks. For a small percentage of the blanks, low-concentration detections above the MDL but below the RL were measured for PCE and TCE.

To monitor extraction efficiency, 5.0 μg of toluene-d8 was spiked into each field sample and quality control (QC) sample Radiello 130 cartridge immediately prior to extraction. The recoveries were evaluated against laboratory limits of 70% to 130%. All surrogate recoveries met the laboratory criterion, and summary statistics are presented in Table A4.

Accuracy of the extraction and analysis step for the target compounds was evaluated by analyzing a laboratory control sample (LCS). An unused Radiello cartridge was spiked with a standard containing 5.0 μg of each compound of interest. The laboratory acceptance criterion for LCS recovery was 70 to 130%. All LCS recoveries met the control limits of 70 to 130%, and summary statistics are presented in Table A5.

Sample precision was evaluated by collecting field duplicates and by analyzing laboratory control sample duplicates (LCSDs). Field duplicates were collected for approximately every 10 field samples, and an LCSD was prepared and analyzed with each sample analytical batch. Because the LCSD was a second cartridge prepared and extracted in the same manner as the LCS, the relative percentage difference (%RPD) represents the precision of the analytical method from extraction through analysis. The method precision data are summarized in Tables A6 and A7. The laboratory acceptance criterion of %RPD < 25% for the field duplicates was met for all compounds except for TCE in one duplicate sample. The LCS/LCSD met the %RPD < 25% except for benzene in two analytical batches and hexane in five analytical batches.

#### 2.4.2. SKC 575 Badge Sampler

A field/trip blank was collected using a blank SKC 575 badge sampler from the media sample batch sent to the field from the laboratory. The blank sampler was opened during preparation of field samples for deployment and then sealed and stored at ambient temperature until sample shipment. A total of 1 field blank was submitted over the duration of the project. In the case of the laboratory blank, a SKC 575 badge sampler was extracted with each analytical batch to measure background from the sorbent and the extraction process. A total of 2 laboratory blanks were analyzed and reported over the duration of the project. To assist in data interpretation, all blank samples and all field sample results were evaluated down to the MDL. The results of the QC samples for the SKC 575 badge samplers are summarized in Tables A8 and A9. Summary statistics were then calculated on the subset of samples with positive detections. Benzene was detected in the field/trip and laboratory blank above the MDL. A positive bias for benzene was anticipated for the 6-month and 1-year SKC575 badge samples due to the blank levels. The average mass of benzene collected on the sorbent for the 6-month and 1-year SKC 575 badge samples was 0.94 and 1.2 ug/m^3^, respectively. No detections of any of the other target analytes were measured in any of the blanks.

Accuracy of the extraction and analysis step for the target compounds was evaluated by analyzing an LCS. An unused SKC 575 badge sampler was spiked with a standard containing 5.0 μg of each compound of interest. The laboratory acceptance criterion for LCS recovery was 70 to 130%. All LCS recoveries met the control limits of 70 to 130%, and summary statistics are presented in Table A10.

Sample precision was evaluated by collecting field duplicates and by analyzing LCSDs. Field duplicates were collected for approximately every 10 field samples, and an LCSD was prepared and analyzed with each sample preparation batch. Because the LCSD was a second badge that was prepared and extracted in the same manner as the LCS, the %RPD represents the precision of the analytical method from extraction through analysis. The method precision is summarized in Tables A11 and A12. The laboratory acceptance criterion of %RPD < 25% was met for all compounds except for benzene, chloroform, and hexane in one field duplicate sample and hexane in two analytical batches.

To monitor extraction efficiency, 5.0 μg of toluene-d8 was spiked into each field sample and QC sample immediately prior to extraction. The recoveries were evaluated against laboratory limits of 70% to 130%. All surrogate recoveries met the laboratory criterion, and summary statistics are presented in Table A13.

#### 2.4.3. WMS^™^

A field/trip blank was collected using a blank WMS^™^ sampler from the media sample batch sent to the field from the laboratory. The blank sampler was opened during preparation of field samples for deployment and then sealed and stored at ambient temperature until sample shipment. A total of 1 field/trip blank was submitted over the duration of the project. Benzene was detected above the MDL but below the reporting limit. No other compounds were detected in the field/trip blank.

For a laboratory blank, a WMS^™^ sampler was extracted with each analytical batch to measure background from the sorbent and the extraction process. A total of 2 laboratory blanks were analyzed and reported over the duration of the project. To assist in data interpretation, all blank samples and all field sample results were evaluated down to the MDL. The results of the field and laboratory blanks for the WMS^™^ samplers are summarized in Tables A14 and A15. Summary statistics were then calculated on the subset of sample with positive detections.

Benzene was the only target analyte detected in the one field/trip and the laboratory blanks above the MDL. The blank level of benzene is significant when evaluating the 6-month and 1-year samples with which it is associated, because the concentrations detected in the samples were less than 10 times the amount detected in the blank. No detections of chloroform, cis-1,2-DCE, hexane, toluene, PCE, or TCE were measured in any of the blanks. To monitor extraction efficiency, 5.0 μg of toluene-d8 was spiked into each field sample and QC sample WMS vial immediately prior to extraction. The recoveries were evaluated against laboratory limits of 70% to 130%. All surrogate recoveries met the laboratory criterion, and summary statistics are presented in Table A16.

Accuracy of the extraction and analysis steps for the target compounds was evaluated by analyzing an LCS. An unused WMS^™^ sampler was spiked with a standard containing 5.0 μg of each compound of interest. The laboratory acceptance criterion for LCS recovery was 70 to 130%. All LCS recoveries met the control limits of 70 to 130%, and summary statistics are presented in Table A17.

Sample precision was evaluated by collecting field duplicates and by analyzing LCSDs. Field duplicates were collected for approximately every 10 field samples, and an LCSD was prepared and analyzed with each sample preparation batch. Because the LCSD was a second cartridge prepared and extracted in the same manner as the LCS, the %RPD represents the precision of the analytical method from extraction through analysis. The method precision is summarized in Tables A18 and A19. The laboratory acceptance criterion of %RPD < 25% was met for all compounds except for hexane in one field duplicate and one LCSD.

The WMS^™^ sample concentrations were adjusted for uptake rate variations due to temperature as recorded onsite by an Onset HOBO^®^ data logging system placed on each wooden sample rack. All measurements were made in accordance with an approved quality assurance project plan. Although some deviations were identified, the deviances did not affect the interpretation of our results [17].

## 3. Results and Discussion

Concentrations of benzene, chloroform, hexane, PCE, toluene, and TCE ranged from 0.4 to 2.3, 0.1 to 4.0, 0.23 to 2.6, 0.1 to 22, 0.44 to 6.0, and 0.1 to 2.7 μg/m^3^, respectively. Several high PCE concentrations were identified when first initiating research at the Indianapolis test house. Removal of the highest PCE concentration samples led to typical PCE concentrations ranging from 0.1 to 2.2 μg/m^3^. Results below the detection limit in the individual weekly samples used for comparison to the longer duration samples were most common for TCE; thus, they introduced the greatest uncertainty in the evaluation of passive sampler performance for TCE.

### 3.1. Phase 1—Radiello^®^ 130 Passive Sampler Duration Testing

If the effective uptake rate decreased with increasing sampling duration, the mean %Bias would be expected to be less for the shorter exposure periods than for the longer exposure periods (i.e., the longer exposure period samples would consistently underestimate the actual vapor concentration). In fact, this was the case. The mean %Bias was generally less for the shorter exposure periods than longer exposure periods for all six VOCs, with the exception of biweekly and monthly PCE and biweekly and semiannual toluene which had negative mean %Bias values (Table 1). These results are in agreement with the findings of Oury et al. [8]. When comparing the mean %Bias of the weekly samples to the mean %Bias of the biweekly samples, all six VOCs were within the acceptance limit of ±30%. As the comparison period lengthened to weekly versus monthly, quarterly, and semiannually, the number of compounds outside the acceptance criterion increased from one to three and then to four, respectively, indicating decreased sampler uptake rates with increased exposure time. Only toluene and PCE mean %Bias values were within the acceptance criterion for a full 1 year after initial sampler deployment. Chloroform and hexane had the overall greatest standard deviations with respect to the average of %Bias across all comparison periods, while toluene and PCE always had the smallest standard deviations.

**Figure 2.**
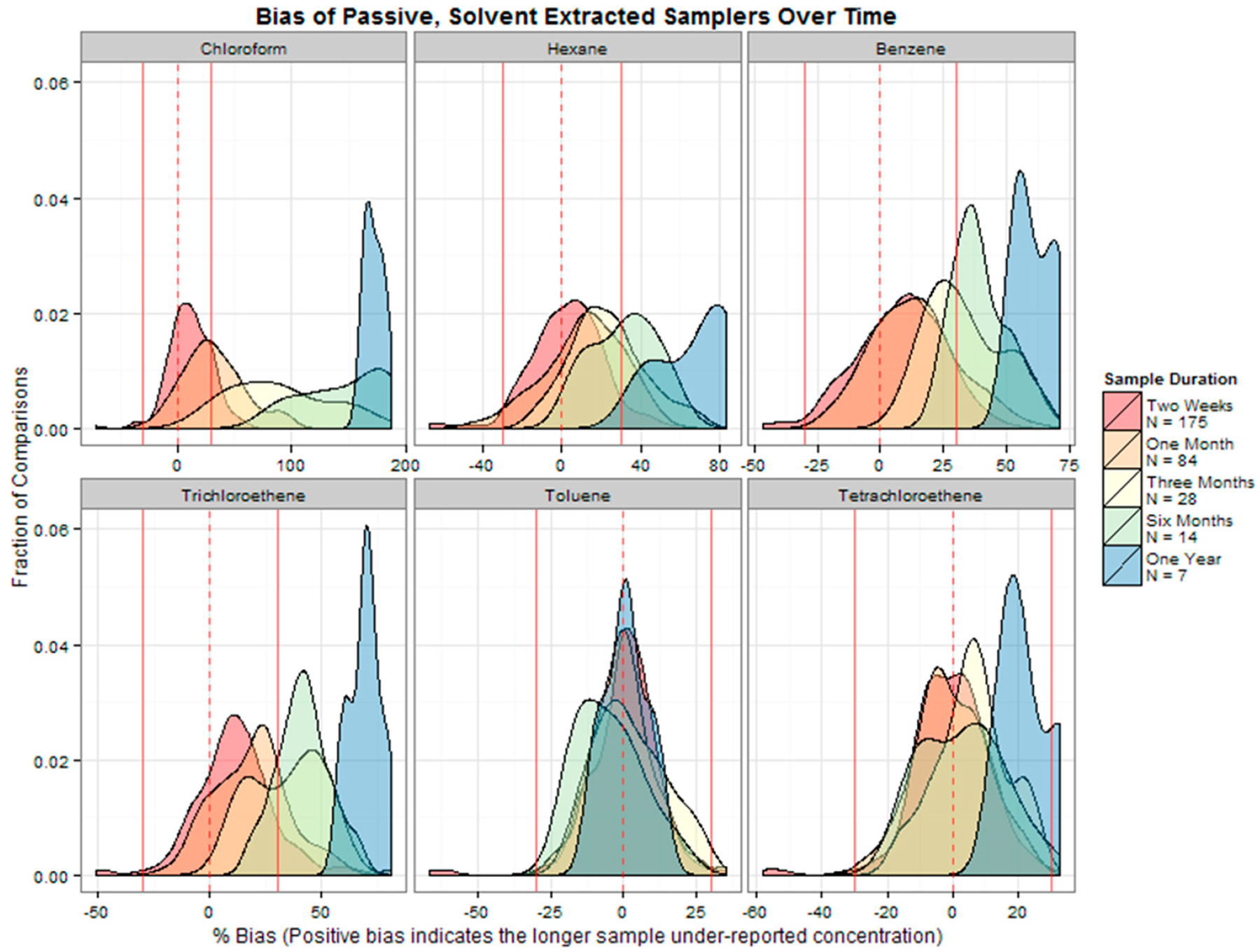
Kernel densities of %Bias for VOCs in the Indianapolis test house. The dotted red line represents a %Bias of zero, and the solid red lines bracket the study acceptance criterion of ± 30%.

To readily visualize the effects of longer-term exposure durations on the measurement of VOC concentrations, kernel density plots were created (Figure 2). The kernel density plots are approximations to the data probability distribution and are affected by sample size (*n*); therefore, caution should be used during interpretation when *n* < 30, as was the case for phase 2. For example, when examining the kernel density plot for chloroform, the peak after 2 weeks was within the ±30% acceptance criterion (i.e., between the solid red lines). After exposure for 1 month, the peak was nearly +30%, indicating that 1 month is about the limit for using a Radiello^®^ 130 passive sampler with the white diffusive body at the relevant chloroform concentrations in the Indianapolis test house. This sorption capability limit for chloroform is supported numerically where the mean %Bias was 31.39%, just slightly above the acceptance criterion (Table 2). As the exposure period extended beyond 1 month, the peaks for 3 months, 6 months, and 1 year increasingly showed greater positive %Bias, well outside the acceptance criterion.

In contrast to chloroform, toluene and PCE %Bias values were within the acceptance criterion throughout the entire year, although a positive shift was identified for PCE with the 1-year exposure period (Figure 2). Hexane and benzene %Bias values appear to be acceptable for up to 3-month exposure periods, although numerically, the benzene mean %Bias was slightly above the acceptance criterion at 33.05% (Table 2). This apparent conflict indicates that the Radiello^®^ 130 passive sampler with the white diffusive body was at its sorption capability limit at the relevant benzene concentrations in the Indianapolis test house at the end of the 3-month exposure period. A bimodal kernel density estimation was noted for TCE with the 3-month exposure period, with the first peak being within the acceptance limits while the second peak was outside the acceptance limits (Figure 2). The quarterly mean %Bias value for TCE was 34.17% (Table 2), indicating an approximate 1-month exposure limit for the Radiello^®^ 130 passive sampler with the white diffusive body.

**Figure 3.**
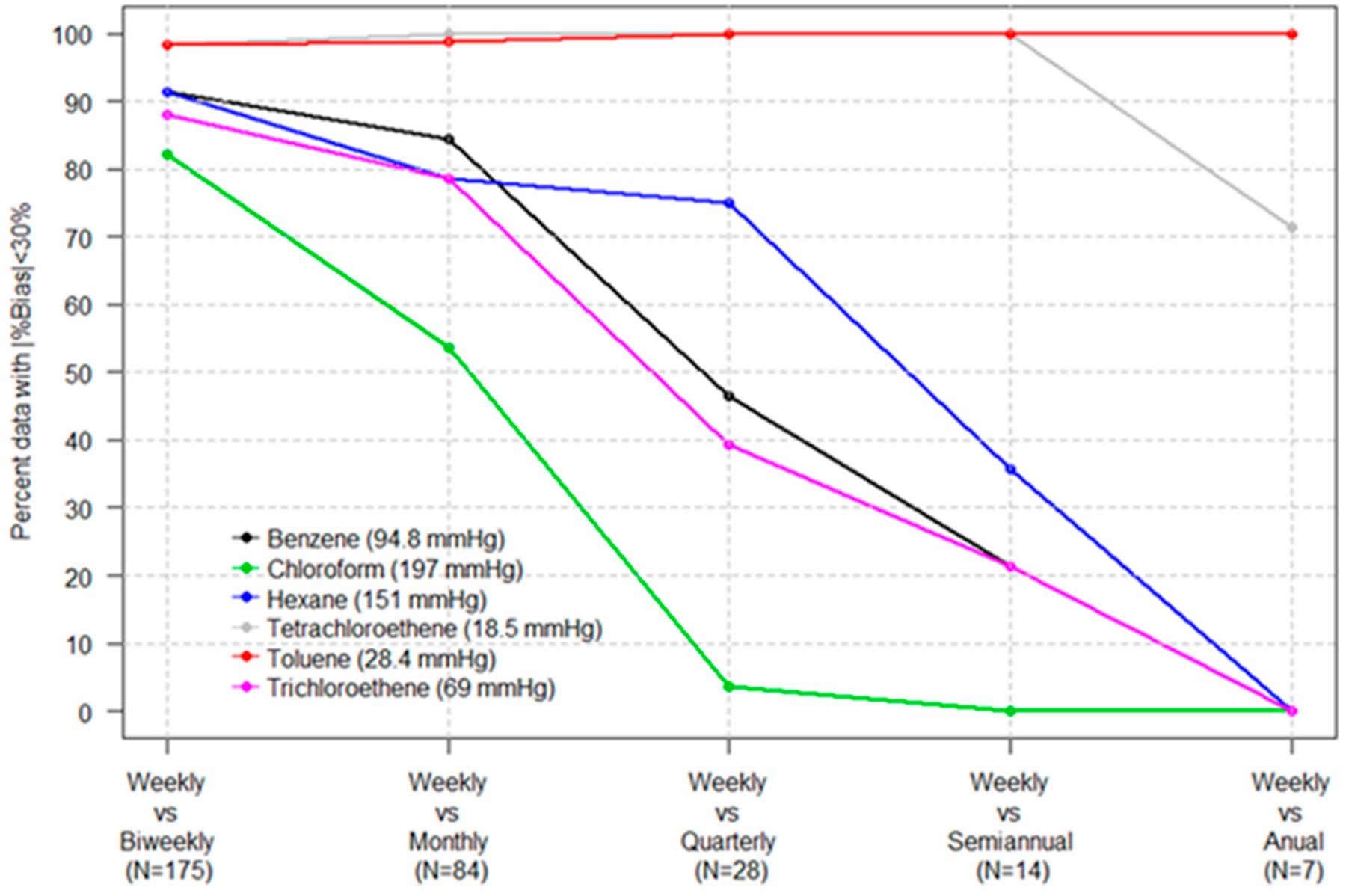
The effect of vapor pressure on sorbent performance.

In general, the phase 1 %Bias data suggest that the stability of the uptake rate is a function of the compound’s volatility as measured by vapor pressure. The VOCs with higher vapor pressures shifted toward a positive bias at shorter exposure intervals than VOCs with lower vapor pressures. The maximum sampling exposure period, defined by meeting the mean %Bias acceptance criterion, follows the expected order from shortest to longest based on the compound’s volatility. The most volatile compound, chloroform, was the first VOC to exceed the average ±30% criterion, and exposure intervals could not be acceptably extended beyond 4 weeks. Benzene and TCE were similar in their volatility and essentially showed comparable performance in their %Bias data, exceeding the criterion at the quarterly exposure measurement. The two least volatile compounds, toluene and PCE, demonstrated a uniform uptake rate over the course of a year. Hexane was the exception to the volatility order, with steady uptake rates extending through the 3-month exposure period. Figure 3 shows that as the period of measurements increases from biweekly to annually, the number of %Bias satisfying the equivalency rate decreases for all VOCs except toluene. While these results may be generally interpreted in terms of volatility, this is not a perfect explanation, and it is likely that solubility in CS_2_ and sorption or partition coefficients into the sorbent may also play a role.

The charcoal sorbent cartridge in the Radiello^®^ 130 passive sampler had sufficient capacity for a 52-week duration with mass loadings onto the cartridges well under the manufacturer’s recommended limit of 80 mg. The sum of the target VOC masses collected on the sampler for the year-long samples was less than 0.2 mg for all the samples, and the total mass on the samplers was estimated to be generally less than 1 mg. If VOC concentrations are significantly higher than what was measured at the Indianapolis test house, then additional considerations should be made regarding extending the sampling duration to ensure sampler capacity is not exceeded.

### 3.2. Phase 2—Passive Sampler 6-Month and 1-Year Testing

Phase 2 exposed the WMS^™^, SKC 575 with secondary diffusive cover, and Radiello^®^ 130 samplers with yellow diffusive bodies for 6- and 12-month exposure periods in the Indianapolis test house. Radiello^®^ 130 passive samplers with white diffusive bodies were collocated with the other samplers and exposed for 1-week periods. Overall, reducing the uptake rate improved performance of passive samplers over the long-term durations. While sampler performance was evaluated against the ±30% acceptance criterion as compared to the weekly average of the Radiello^®^ 130 passive samplers with the white diffusive bodies, the average VOC concentrations from three long-term samplers were typically within a factor of two of the weekly average VOC concentration over the same exposure periods (Tables 3 and 4). Results for TCE were not included in the data evaluations for phase 2 of the study because the majority of detected concentrations were at or below the reporting limit.

The WMS^™^ had the lowest published uptake rate; however, it generally yielded the lowest concentration of the three passive samplers tested and was typically lower than the average 1-week Radiello^®^ 130 passive sampler with the white diffusive body concentration for the same exposure period. The WMS^™^ had acceptable %Bias values for two out of three locations in the Indianapolis test house for benzene, toluene, and hexane when exposed for 6 months. The %Bias values continued to be within acceptance limits for toluene and hexane after 1 year. The WMS had %Bias values exceeding the 30% acceptance criteria at two of the three locations for chloroform and all three locations for PCE for the 6-month exposure, as well as all locations for chloroform and PCE for the 1-year exposure. It appears that the charcoal-based sorbent bed in the WMS^™^ may be better suited for the sampling of petroleum hydrocarbons than chlorinated solvents over these longer exposure periods.

The SKC 575 badge sampler had the next highest uptake rate and typically had the highest concentration relative to the other samplers and the weekly average, most specifically for toluene, of the Radiello^®^ 130 white diffusive body sampler. After the 6-month exposure, the %Bias values for the SKC 575 sampler were nearly always within the ±30% acceptance limit except for benzene. These results were similar to findings by Oury et al. [8], who attributed the loss of benzene with longer exposure times to back diffusion from the sampler. In contrast, after 12 months, only hexane %Bias values were acceptable at all three sampling locations.

Uptake rates for the Radiello^®^ 130 passive sampler with yellow diffusive bodies were estimated using the effective geometric constant of the yellow diffusive body and resulted in concentrations that were relatively accurate based on comparison to the weekly average concentrations. For chloroform and PCE, 6-month exposure times were always within the acceptance limits. Benzene was almost always within the acceptance limits after 6 months of exposure. After the 1-year exposure period, chloroform showed a strong positive bias, likely due to its weak adsorption to the sorbent bed and subsequent back diffusion. Interestingly, after 1 year, only hexane had acceptable %Bias values in two of the three sampling locations. Similarly, the WMS^™^ and SKC 575 passive samplers also showed a marked decrease in the effective uptake rate and subsequent elevated %Bias values for chloroform when exposed for 1 year.

## 4. Conclusions

### 4.1. Phase 1—Radiello^®^ 130 Passive Sampler Duration Testing

The radial-style, charcoal-based passive sampler performance over exposure periods from 1 week to 1 year was dependent on the target compound. The charcoal-based sorbent cartridge had sufficient capacity for the 1-year exposure period with estimated loadings of less than 1 mg, which were well below the manufacturer’s recommended loading limit of 80 mg. Hence, sorptive capacity of the passive sampler was not a factor influencing the results. All VOCs showed excellent agreement between the mean %Bias of the 1-week and the 2-week samples, suggesting uniform uptake rates over this comparison period within the ±30% acceptance limit. The mean %Bias for chloroform was just outside the acceptance criterion after the 4-week exposure period. TCE uptake was constant up to an approximate 1-month exposure period but failed to meet the acceptance criterion after a 3-month exposure period. Benzene and hexane exhibited generally stable uptake rates through the monthly exposure period but failed to meet the acceptance criterion for the quarterly, semi-annual, and annual exposure periods. Toluene and PCE continued to demonstrate stable uptake rates after the quarterly, semiannual, and annual exposure periods and had %Bias values within the ±30% acceptance criterion.

### 4.2. Phase 2—Passive Sampler 6-Month and 1-Year Testing

The WMS^™^, SKC 575, and Radiello^®^ 130 passive samplers with yellow diffusive bodies were exposed for 6-month and 1-year periods, and the resultant VOC concentrations were compared to the average VOC concentrations from the Radiello^®^ 130 passive samplers with white diffusive bodies that had been exposed for 1-week periods. Overall, the lower uptake rate associated with these three passive samplers improved performance (i.e., decreased the %Bias values) over the longer exposure periods. The SKC 575 sampler and the Radiello^®^ 130 passive sampler with the yellow diffusive body generally produced acceptable results over the 6-month exposure period, while the WMS^™^ sampler with a charcoal-based sorbent bed appeared to be more suited for the collection of the petroleum hydrocarbons than the chlorinated solvents. After the 1-year exposure period, the overall passive sampler performances were inconsistent, except for hexane, which was acceptable for all three passive samplers. While passive sampler performance was evaluated against the ±30% acceptance criterion as compared to the weekly average of the Radiello^®^ 130 passive samplers with the white diffusive bodies, the three long-term samplers evaluated had VOC concentrations that were typically within a factor of two of the weekly average concentration.

In general, the results suggest that all three samplers can produce acceptable results over long periods; however, customizing each sampler to site-specific conditions, for example, by empirically deriving uptake rates for specific environments, sampling durations, and analytes, may be necessary to optimize performance during a long-term study. If VOC concentrations are significantly higher than those measured at our test site, additional considerations should be made before extending the exposure period to ensure sampler sorptive capacity is not exceeded. These results show that extended sampling times with passive samplers can be a cost-effective tool for estimating chronic or sub-chronic exposure for selected VOCs.

## Figures and Tables

**Table 1.** Specifications for SKC 575, WMS, and Radiello 130 with white/yellow diffusive bodies.

Sampler	SKC 575	WMS	Radiello 130, White Body	Radiello 130, Yellow Body
**Type and Mass of Sorbents**	Anasorb CSC, 350 mg	Anasorb 747^®^, 165 mg	Activated charcoal (30–50 mesh), 530 ± 30 mg	Activated charcoal (30–50 mesh), 530 ± 30 mg
**Diffusion Media**	Nylon	polydimethylsiloxane (PDMS) membrane	Microporous polyethylene 1.7 mm thick and average porosity 25 ± 5 μm. Diffusive path length is 18 mm.	Microporous polyethylene 5 mm thick and average porosity 10 ± 2 μm. Diffusive path length is 150 mm.
**Dimensions**	Diameter: 1.4 in (3.5 cm); length (including clip): 2.5 in (6.3 cm); depth: 0.6 in (1.5 cm)	1.8 mL crimp top glass vial	60 mm length and 4 mm diameter	60 mm length and 4 mm diameter
Sample uptake Rates (mL/min)				
**Benzene**	16.0^a^	2.2^b^	80^c^	27^d^
**Hexane**	14.3^a^	1.5^b^	66^c^	23^d^
**TCE**	14.9^a^	2.6^b^	69^c^	24^d^
**PCE**	13.1^a^	5.4^b^	59^c^	20^d^

a [18];

b [19];

c [20];

d Calculated from sample uptake rate for Radiello 130 with white diffusive body times the ratio of the geometric constants of the yellow diffusive body to the white diffusive body (ratio = 0.34256) as per [21].

**Table 2.** Summary statistics for %Bias by exposure period and VOC.

Exposure Periods	*n* ^a^	%Bias^b^	Standard Deviation
**Weekly vs. Biweekly**			
Benzene	175	7.74	16.98
Chloroform	175	11.15	18.55
Hexane	175	2.75	18.85
Tetrachloroethylene	175	−0.44	11.78
Toluene	175	−0.02	12.13
Trichloroethylene	175	10.98	17.23
**Weekly vs. Monthly**			
Benzene	84	13.39	16.80
Chloroform	84	31.39	26.89
Hexane	84	11.33	20.00
Tetrachloroethylene	84	−0.47	10.89
Toluene	84	0.03	10.68
Trichloroethylene	84	19.09	17.65
**Weekly vs. Quarterly**			
Benzene	28	33.05	14.83
Chloroform	28	88.29	43.58
Hexane	28	23.05	19.29
Tetrachloroethylene	28	5.88	10.82
Toluene	28	2.37	12.03
Trichloroethylene	28	34.17	15.94
**Weekly vs. Semiannual**			
Benzene	14	39.48	10.89
Chloroform	14	147.00	36.58
Hexane	14	31.61	16.71
Tetrachloroethylene	14	2.93	12.44
Toluene	14	−4.84	11.41
Trichloroethylene	14	61. 7	11.91
**Weekly vs. Annual**			
Benzene	7	60.81	7.91
Chloroform	7	172.30	8.45
Hexane	7	67.19	18.10
Tetrachloroethylene	7	22.37	7.54
Toluene	7	0.80	7.77
Trichloroethylene	7	69.30	7.25

a— *n* = number of samples.

b— yellow shaded boxes within the ±30% acceptance limit.

**Table 3.** Indianapolis test house 6-month passive sampler concentration comparison (μg/m^3^).

	VOC Concentration (μg/m^3^)				%Bias (Against Weekly Avg.)		
Location	WMS^™^	SKC 575^a^	Radiello^®^ 130 Yellow	Radiello^®^ 130 White Weekly Average	WMS^™^	SKC 575	Radiello^®^ 130 Yellow
**Benzene**							
422 Base S	0.60	0.58	0.52	0.87	37%	41%	50%
422 First	0.65	0.66	0.71	0.83	24%^b^	22%	16%
420 Base S	0.63	0.60	0.68	0.83	27%	33%	20%
**Toluene**							
422 Base S	0.79	1.2	1.2	0.98	21%	−22%	−20%
422 First	0.71	1.2	1.5	0.98	32%	−22%	−42%
420 Base S	0.84	1.2	1.4	0.95	12%	−20%	−38%
**Hexane**							
422 Base S	0.35	0.40	0.52	0.50	−30%	−21%	4%
422 First	0.26	0.52	0.79	0.52	−50%	−1%	52%
420 Base S	0.61	0.59	0.83	0.49	24%	20%	69%
**Chloroform**							
422 Base S	0.47	0.94	0.60	0.60	24%	−44%	0%
422 First	0.15	0.40	0.35	0.32	72%	−22%	−9%
420 Base S^c^	0.10	0.16	0.13	0.17	52%	4%	27%
**Tetrachloroethylene**							
422 Base S	0.61	1.1	0.96	0.94	43%	−12%	−2%
422 First	0.26	0.52	0.62	0.47	58%	−11%	−28%
420 Base S	0.14	0.23	0.27	0.22	44%	−4%	−20%

a— Used published outdoor uptake rates as more representative of basement indoor duplex conditions.

b— Yellow boxes indicate value within ±30% acceptance limit.

c— Percent bias calculations influenced by high number of nondetects in Radiello 130^®^ white passive sampler weekly averages.

**Table 4.** Indianapolis test house 1-year passive sampler concentration comparison (μg/m^3^).

	VOC Concentration (μg/m^3^)				%Bias (Against Weekly Avg.)		
Location	WMS^™^	SKC 575^a^	Radiello^®^ 130 Yellow	Radiello^®^ 130 White Weekly Average	WMS^™^	SKC 575	Radiello^®^ 130 Yellow
**Benzene**							
422 Base S	0.36	0.49	0.47	0.88	84%	57%	61%
422 First	0.35	0.52	0.56	0.82	80%	44%	38%
420 Base S	0.35	0.48	0.52	0.75	73%	43%	36%
**Toluene**							
422 Base S	1.0	1.5	1.6	1.2	15%^b^	−27%	−32%
422 First	0.93	1.5	1.8	1.0	6%	−43%	−58%
420 Base S	0.99	1.6	1.7	1.1	12%	−34%	−41%
**Hexane**							
422 Base S	0.62	0.50	0.55	0.56	−10%	13%	2%
422 First	0.65	0.44	0.67	0.51	−23%	15%	−26%
420 Base S	0.40	0.64	0.75	0.51	24%	−23%	−38%
**Chloroform**							
422 Base S	0.17	0.17	0.019	0.50	99%	98%	185%
422 First	0.10	0.14	0.022	0.31	102%	75%	173%
420 Base S^c^	0.048	0.053	0.014	0.10	73%	64%	152%
**Tetrachloroethylene**							
422 Base S	0.33	0.57	0.54	0.88	91%	43%	48%
422 First	0.16	0.31	0.33	0.46	97%	38%	33%
420 Base S	0.094	0.17	0.18	0.19	69%	14%	7%

a— Used published outdoor uptake rates as more representative of basement indoor duplex conditions.

b— Yellow boxes indicate value within ±30% acceptance limit.

c— Percent bias calculations influenced by high number of nondetects in Radiello^®^ 130 white passive sampler weekly averages.

## Data Availability

The original data presented in the study are openly available at DATA.gov or DOI: 10.23719/1528744.

## Appendix A

**Table A1.** Indoor air passive field blank summary—Radiello^®^ 130.

Analyte	RL (μg)	Number of Field Blanks			% of Field Blanks with Detections	Mean Blank Conc. (μg)	Std Dev (μg)	Min (μg)	Max (μg)
		Analyzed	Conc. > RL	RL > Conc. > MDL					
Benzene	0.4	47	0	38	81%	0.11	0.042	0.040	0.18
Chloroform	0.1	47	0	0	0%	NA	NA	NA	NA
cis-1,2-DCE	0.1	47	0	0	0%	NA	NA	NA	NA
Hexane	0.1	47	4	9	28%	0.099	0.091	0.033	0.35
PCE	0.1	47	0	2	9%	0.032	0.020	0.0067	0.049
Toluene	0.1	47	1	21	47%	0.040	0.036	0.014	0.17
TCE	0.1	47	0	5	11%	0.015	0.0093	0.0064	0.031

RL = reporting limit; MDL = method detection limit; Std Dev = standard deviation; NA = not applicable.

**Table A2.** Indoor air passive trip blank summary—Radiello^®^ 130.

Analyte	RL (μg)	Number of Trip Blanks			% of Trip Blanks with Detections	Mean Blank Conc. (μg)	Std Dev (μg)	Min (μg)	Max (μg)
		Analyzed	Conc. > RL	RL > Conc. > MDL					
Benzene	0.4	22	0	20	91%	0.10	0.039	0.042	0.16
Chloroform	0.1	22	0	0	0%	NA	NA	NA	NA
cis-1,2-DCE	0.1	22	0	0	0%	NA	NA	NA	NA
Hexane	0.1	22	0	10	45%	0.049	0.012	0.036	0.07
PCE	0.1	22	0	2	9%	0.015	0.009	0.0087	0.022
Toluene	0.1	22	0	18	82%	0.020	0.008	0.012	0.041
TCE	0.1	22	0	4	18%	0.024	0.0159	0.0094	0.043

RL = reporting limit; MDL = method detection limit; Std Dev = standard deviation; NA = not applicable.

**Table A3.** Indoor air passive laboratory blank summary—Radiello^®^ 130.

Analyte	RL (μg)	Number of Lab Blanks			% of Lab Blanks with Detections	Mean Blank Conc. (μg)	Std Dev (μg)	Min (μg)	Max (μg)
		Analyzed	Conc. > RL	RL > Conc. > MDL					
Benzene	0.4	73	0	67	92%	0.12	0.043	0.039	0.22
Chloroform	0.1	73	0	0	0%	NA	NA	NA	NA
cis-1,2-DCE	0.1	73	0	0	0%	NA	NA	NA	NA
Hexane	0.1	73	0	18	25%	0.053	0.019	0.034	0.083
PCE	0.1	73	0	2	3%	0.0081	0.00042	0.0078	0.0084
Toluene	0.1	73	0	52	71%	0.025	0.014	0.012	0.064
TCE	0.1	73	0	4	6%	0.022	0.0068	0.013	0.027

RL = reporting limit; MDL = method detection limit; Std Dev = standard deviation; NA = not applicable.

**Table A4.** Indoor air passive surrogate summary—Radiello^®^ 130.

Parameter	Result
Number of surrogate recoveries measured	1255
Average recovery (%R)	102.8
Standard deviation (%R)	5.9
Minimum recovery (%R)	87
Maximum recovery (%R)	122

**Table A5.** Indoor air passive laboratory control sample (LCS) summary—Radiello^®^ 130.

Analyte	Number of LCS Analyzed	Mean LCS % Recovery	LCS Std Dev (%R)	Min (%R)	Max (%R)
Benzene	73	93	11.0	71	116
Chloroform	73	96	11.5	70	122
cis-1,2-DCE	73	95	8.7	72	121
Hexane	73	101	14.6	71	130
PCE	73	98	9.8	73	125
Toluene	73	94	9.8	73	117
TCE	73	97	8.6	73	118

**Table A6.** Indoor air passive field duplicate summary—Radiello^®^ 130.

Analyte	Number of Field Duplicates Analyzed	Mean	Std Dev. (%RPD)	Min (%RPD)	Max (%RPD)	Number of Exceedances
		%RPD				
Benzene	4	4.8	2.9	1.9	8.4	0
Chloroform	4	6.2	2.5	4.0	11	0
cis-1,2-DCE	4	ND	NA	NA	NA	NA
Hexane	4	14	9.5	0	25	0
PCE	4	5.8	3.8	0	11	0
Toluene	4	5.2	3.2	0	8.7	0
TCE	4	7.8	11	0	27	1

ND = not detected, NA = not applicable.

**Table A7.** Indoor air passive laboratory precision (LCS/LCSD) summary—Radiello^®^ 130.

Analyte	Number of LCSD Analyzed	Mean%RPD	Std Dev. (%RPD)	Min (%RPD)	Max (%RPD)	Number of Exceedances
Benzene	73	9%	8%	0%	29%	2
Chloroform	73	10%	7%	0%	25%	0
cis-1,2-DCE	73	5%	4%	0%	19%	0
Hexane	73	11%	9%	0%	37%	5
PCE	73	5%	4%	0%	19%	0
Toluene	73	5%	4%	0%	19%	0
TCE	73	5%	4%	0%	14%	0

**Table A8.** Indoor air passive field/trip blank summary—SKC 575.

Analyte	RL (μg/m^3^)	Field/Trip Blank (μg/m^3^)
Benzene	0.15	0.098 J
Chloroform	0.039	0.039 U
cis-1,2-DCE	0.034	0.034 U
Hexane	0.041	0.041 U
PCE	0.039	0.039 U
Toluene	0.044	0.044 U
TCE	0.034	0.034 U

J—Estimated value, U—Compound analyzed for but not detected above the reporting limit.

**Table A9.** Indoor air passive laboratory blanks summary—SKC 575.

Analyte	Number of Lab Blanks				% of Lab Blanks with Detections	Mean Blank Conc. (μg/m^3^)	Std Dev (μg/m^3^)	Min (μg/m^3^)	Max (μg/m^3^)
	RL (μg)	Analyzed	Conc. > RL	RL > Conc. > MDL					
Benzene	0.4	2	0	2	100%	0.07	0.04	0.03	0.11
Chloroform	0.1	2	0	0	0%	ND	NA	NA	NA
cis-1,2-DCE	0.1	2	0	0	0%	ND	NA	NA	NA
Hexane	0.1	2	0	0	0%	ND	NA	NA	NA
PCE	0.1	2	0	0	0%	ND	NA	NA	NA
Toluene	0.1	2	0	0	0%	ND	NA	NA	NA
TCE	0.1	2	0	0	0%	ND	NA	NA	NA

ND = not detected, NA = not applicable.

**Table A10.** Indoor air passive LCS samples summary—SKC 575.

Analyte	Number of LCS Analyzed	Mean LCS % Recovery	LCS Std Dev (%R)	Min (%R)	Max (%R)
Benzene	2	91	7	84	98
Chloroform	2	95	2	93	97
cis-1,2-DCE	2	96	1	95	96
Hexane	2	92	14	78	105
PCE	2	103	3	100	105
Toluene	2	101	6	95	107
TCE	2	104	4	100	107

**Table A11.** Indoor air passive field duplicate samples summary—SKC 575.

Analyte	Number of Sample Duplicates Analyzed	Mean	Std Dev. (%RPD)	Min (%RPD)	Max (%RPD)	Number of Exceedances
		%RPD				
Benzene	2	10	5.4	4.2	15	1
Chloroform	2	16	11	4.7	26	1
cis-1,2-DCE	2	ND	NA	NA	NA	NA
Hexane	2	8.6	5.2	3	14	1
PCE	2	0.7	0.7	0	1.3	0
Toluene	2	0	0	0	0	0
TCE	2	6.0	3.1	2.9	9.2	0

ND = not detected, NA = not applicable.

**Table A12.** Indoor air passive laboratory precision (LCS/LCSD) summary—SKC 575.

Analyte	Number of LCSD Analyzed	Mean	Std Dev. (%RPD)	Min (%RPD)	Max (%RPD)	Number of Exceedances
		%RPD				
Benzene	2	12	3.8	7.7	15	0
Chloroform	2	14	2.4	11	16	0
cis-1,2-DCE	2	2.7	1.6	1.0	4.3	0
Hexane	2	41	5.2	36	46	2
PCE	2	2.0	2.0	0.0	4.1	0
Toluene	2	0.9	0.9	0.0	1.9	0
TCE	2	2.0	0.1	1.9	2.0	0

**Table A13.** Indoor air passive surrogate summary—SKC 575.

Parameter	Result
Number of surrogate recoveries measured	15
Average recovery (%R)	109
Standard deviation (%R)	5.8
Minimum recovery (%R)	102
Maximum recovery (%R)	115

**Table A14.** Indoor air passive field/trip blank summary—WMS^™^.

Analyte	RL (μg/m^3^)	Blank Conc. (μg/m^3^)
Benzene	0.36	0.24 J
Chloroform	0.098	0.098 U
cis-1,2-DCE	0.10	0.10 U
Hexane	0.59	0.59 U
PCE	0.036	0.036 U
Toluene	0.045	0.045 U
TCE	0.058	0.058 U

RL = reporting limit; J = estimated value. U = Compound analyzed for but not detected above the reporting limit.

**Table A15.** Indoor air passive laboratory blank summary—WMS^™^.

Analyte	Number of Lab Blanks				% of Lab Blanks with Detections	Mean Blank Conc. (ug/m^3^)	Std Dev (ug/m^3^)	Min (ug/m^3^)	Max (ug/m^3^)
	RL (ug/m^3^)	Analyzed	Conc. > RL	RL > Conc. > MDL					
Benzene	0.36	2	0	1	50%	0.27 J	NA	0.27	0.27
Chloroform	0.10	2	0	0	0%	0.10 U	NA	NA	NA
cis-1,2-DCE	0.10	2	0	0	0%	0.10 U	NA	NA	NA
Hexane	0.59	2	0	0	0%	0.59 U	NA	NA	NA
PCE	0.036	2	0	0	0%	0.036 U	NA	NA	NA
Toluene	0.045	2	0	0	0%	0.045 U	NA	NA	NA
TCE	0.058	2	0	0	0%	0.058 U	NA	NA	NA

RL = reporting limit; MDL = method detection limit; Std Dev = standard deviation; J—Estimated value, U—Compound analyzed for but not detected above the reporting limit; NA = not applicable.

**Table A16.** Indoor air passive surrogate summary—WMS^™^.

Parameter	Result
Number of surrogate recoveries measured	18
Average recovery (%R)	108
Standard deviation (%R)	5.3
Minimum recovery (%R)	102
Maximum recovery (%R)	116

**Table A17.** Indoor air passive laboratory control sample (LCS) summary—WMS^™^.

Analyte	Number of LCS Analyzed	Mean LCS % Recovery	LCS Std Dev (%R)	Min (%R)	Max (%R)	Number of Exceedances
Benzene	2	92	9	83	101	0
Chloroform	2	94	1	93	94	0
cis-1,2-DCE	2	98	2	96	100	0
Hexane	2	88	6	82	93	0
PCE	2	100	2	98	101	0
Toluene	2	102	3	99	105	0
TCE	2	106	1	105	107	0

**Table A18.** Indoor air passive field duplicates summary—WMS^™^.

Analyte	Number of Field Duplicates Analyzed	Mean	Std Dev. (%RPD)	Min (%RPD)	Max (%RPD)	Number of Exceedances
		%RPD				
Benzene	2	3.8	1	2.7	4.9	0
Chloroform	2	7.3	2	5.7	8.9	0
cis-1,2-DCE	2	ND	NA	NA	NA	NA
Hexane	2	36	21	16	57	1
PCE	2	1.5	1.5	0	3.0	0
Toluene	2	0.6	0.6	0	1.3	0
TCE	2	2.7	2.7	0	5.4	0

ND = not detected, NA = not applicable.

**Table A19.** Indoor air passive laboratory precision (LCS/LCSD) summary—WMS^™^.

Analyte	Number of LCSD Analyzed	Mean	Std Dev. (%RPD)	Min (%RPD)	Max (%RPD)	Number of Exceedances
		%RPD				
Benzene	2	10	5	1.9	14	0
Chloroform	2	11	3	8.2	14	0
cis-1,2-DCE	2	2	0	1.0	2	0
Hexane	2	25	17	7.8	42	1
PCE	2	2	1	1.0	2	0
Toluene	2	1	1	0.0	2	0
TCE	2	0	0	0.0	1	0

## Abbreviations

The following abbreviations are used in this manuscript:

VOCs: volatile organic compounds
VI: vapor intrusion
TCE: trichloroethylene
PCE: perchloroethylene
WMS^™^: Waterloo Membrane Sampler^™^
HVAC: heating, ventilation, and air conditioning
RSD: relative standard deviation
BTEX: benzene, toluene, ethylbenzene, and xylene
MDL: method detection limit
GC/MS: gas chromatography–mass spectrometry
TD: thermal desorption
RL: reporting limit
cis-1,2-DCE: cis-1,2-dichloroethene
US EPA: US Environmental Protection Agency
L: liters
LCS: laboratory control sample
%Bias: relative percent difference
QC: quality control
LCSDs: laboratory control sample duplicates
CS_2_: carbon disulfide
